# Demographic and Cardiovascular Risk Factors Associated with Drug Use in Truck Drivers in the State of São Paulo, Brazil: A Cross-Sectional Study

**DOI:** 10.3390/ijerph18094927

**Published:** 2021-05-06

**Authors:** Mariana Moura Pereira, Antonio de Padua Mansur, Julio Yoshio Takada, Vilma Leyton

**Affiliations:** 1 Oscar Freire Institute, University of Sao Paulo Medical School, São Paulo 05405-150, Brazil; ma_mpereira@hotmail.com (M.M.P.); vileyton@gmail.com (V.L.); 2 Heart Institute (InCor), University of Sao Paulo Medical School, São Paulo 05403-000, Brazil; jyt@bol.com.br

**Keywords:** illicit drugs, risk factors, smoking, alcohol drinking, toxicology

## Abstract

The aim of the study is to analyze the association between risk factors for the health of truck drivers and previous use of illicit drugs. A cross-sectional study examined the data from 2071 truck drivers between 2010 and 2016. Demographic variables, risk factors for cardiovascular disease (CVD) and the use of illicit drugs were analyzed. The stepwise logistic regression model was used for the adjusted analysis. The dependent variable was the previous use of illicit drugs, and independent variables were those with *p* < 0.1 at a bivariate analysis. The average age of the truck drivers was 42.27 ± 11.07 years, and the previous use of illicit drugs was reported or detected in 388 (18.7%) drivers. Compared to non-users, drug users were younger (37.25 ± 9.45 vs. 43.43 ± 11.1 years; *p* < 0.001) and single (43.3% vs. 28.4%; *p* < 0.001). The independent variables for illicit drugs were age (OR = 0.93 (95% CI: 0.91–0.95; *p* < 0.001)), smoking (OR = 2.18 (95% CI: 1.39–3.44; *p* = 0.001)), alcohol consumption (OR = 1.626 (95% CI: 1.06–2.49; *p* = 0.026)) and driving hours per day (OR = 1.08 (95% CI: 1.01–1.15; *p* = 0.012)). Users of illicit drugs had multiple risk factors for CVD and traffic accidents.

## 1. Introduction

In Brazil, the road sector was responsible for 61% of cargo transportation and 36.2% of the GDP in 2014 [1]. Cargo transportation is one of the sectors that generates more jobs but is also associated with many fatal traffic accidents [2]. Professional drivers are often exposed to overtime work, irregular sleep patterns and short delivery times. These factors contribute strongly to a higher incidence of chronic non-communicable diseases (NCDs) and the occurrence of traffic accidents [3,4,5]. 

A recent study showed an association between sleepiness and metabolic syndrome, which resulted in an increase in traffic accidents [4]. Poor eating habits, lack of physical activity, obesity and irregular sleep patterns lead to metabolic syndrome and obstructive sleep apnea and, consequently, excessive daytime sleepiness, which is also an important risk factor for accidents. In truck drivers, lifestyle was associated with a higher incidence of heart disease, obesity, sleep disorders and diabetes, and these diseases increase the risk of road accidents [5]. In addition, psychoactive substances have also been used by drivers to alleviate fatigue and sleepiness resulting from long distance trips, driving at night, having short rest periods and few hours of sleep [6,7,8,9,10,11]. Regarding the relationship between health risk factors and drug use, a study conducted with young people between 15 and 35 years old showed that illicit drugs have an important effect on sudden cardiac death [12]. The study showed that illicit drugs, mainly cocaine, amphetamine and marijuana, were detected in 29 young drivers who died by sudden cardiac death among the 98 drivers with positive toxicology for drugs [12]. Substances toxic to the heart have also been found to be present, mainly in individuals who died from chronic or acute ischemic heart disease [13]. There are also reports that cocaine use and abuse are associated with an increased risk of cardiovascular complications, such as systemic arterial hypertension (SAH), coronary artery spasm, arrhythmias, acute myocardial infarction (MI), cardiomyopathy and atherosclerotic coronary artery disease [13]. In turn, tetrahydrocannabinol (THC), the main active component of marijuana, causes an acute dose-dependent increase in blood pressure (BP) and heart rate. A review explored a series of studies on the topic, and there was evidence to suggest that increasing the frequency of marijuana use increases the risk of cardiac arrhythmias and MI [14]. Marijuana contaminants, such as acetaldehyde, ammonia, and benzene, can also play a role in the development of life-threatening cardiovascular diseases [14]. Tobacco use also occurs frequently among marijuana users [14,15,16,17]. Acute stroke has also been associated with marijuana, cocaine and amphetamine use [16]. Marijuana use by young adults was associated with a 17% increase in stroke risk [17]. 

The precarious working conditions were associated with a worse lifestyle that favors NCDs and the use of illicit drugs; these are important risk factors for traffic accidents. Nevertheless, little is known about the demographic, occupational and risk factors for NCD in truck drivers that use illicit drugs. The aim of this study was to analyze the relationship between the previous use of illicit drugs by Brazilian truck drivers and health risk factors among these workers.

## 2. Materials and Methods

### 2.1. Study Participants

This is a cross-sectional cohort and a simple random sampling study that analyzed 2071 truck drivers who traveled on highways in the State of São Paulo from 2010 to 2016. Drivers were randomly stopped to participate in the “Health Commands on the Roads” program promoted by the Federal Highway Police, which aims to promote the health of truck drivers on federal highways [18]. The purpose of the program is to provide guidance to professional drivers regarding good health habits for the prevention of diseases and traffic accidents, as well as to perform medical examinations to detect risk factors for chronic diseases. The participation of the drivers was voluntary, and participants were included in the study after signing an informed consent form. The exclusion criteria were distances smaller than 100 Km, a small utility-service truck vehicle, individuals with questionnaires lacking essential data for analysis and not signing the informed consent form.

### 2.2. Data Collection

Drivers were asked about demographic data (age, sex, marital status, education level and socioeconomic level), occupational data (type of job, time in the profession, number of hours worked per day, type of journey and sleepiness), risk factors for NCDs (smoking, dyslipidemia, diabetes, high blood pressure, obesity and sleepiness) and the use of illicit drugs at least once while working as a truck driver (crack, cocaine, marijuana, synthetic psychoactive drugs and amphetamine). Lifestyle was assessed in a qualitative way and considered sedentary, according to the report of the absence of any additional physical activity unrelated to the regular workday. The Epworth Sleepiness Scale was used to assess sleepiness, with a cutoff of ≤10 for non-drowsiness and >10 for sleepiness [19]. Obesity was defined according to body mass index (BMI = weight (kg)/height (m^2^)) as follows: normal weight (BMI ≥ 18.5 and <25), overweight (BMI ≥ 25 and <30) and obesity (BMI ≥ 30) [20]. SAH was diagnosed according to values of systolic blood pressure >140 mmHg and diastolic blood pressure >90 mmHg or if the participant used antihypertensive drugs [20]. Dyslipidemia was diagnosed in individuals with total cholesterol ≥240 mg/dL, triglycerides ≥150 mg/dL, LDL-cholesterol (LDL) ≥130 mg/dL, or statin use [20]. Diabetes was diagnosed in individuals with fasting glucose ≥126 mg/dL, casual blood glucose ≥200 mg/dL or if hypoglycemic agents were used [20]. The percentage of body fat was calculated using the following formula: %body fat = 495/(1.0324 − 0.19077(log (waist-neck)) + 0.15456(log(height))) − 450(log10) [21]. The circumferences of the neck and waist were considered above the normal limit if the values were ≥40 cm and ≥109 cm, respectively [20]. Alcohol consumption, involvement in traffic accidents and the transport of dangerous cargo were assessed via direct questions with a positive or negative answer. The distance traveled took into account the city of departure and the city of the destination and was defined in km in the drivers’ reports.

### 2.3. Toxicological Analysis

Urine and oral fluid were also collected from the drivers for toxicological testing. Quick tests were applied at the site to measure blood glucose, cholesterol and triglyceride levels. Samples for toxicological analysis were collected in the field and analyzed in the laboratory. The following drugs and/or their metabolites were researched: cocaine, cannabis and amphetamine. Immunoassay screening tests were carried out, and the positive results were confirmed by mass spectrometry using previously described methodologies [8,9,10].

### 2.4. Statistical Analysis

Drivers were divided into two groups: those who reported never having used illicit drugs and those who reported having used illicit drugs at least once in their lives. For continuous variables, the analysis was performed by observing the minimum and maximum values and calculating means and standard deviations. For categorical variables, absolute and relative frequencies were calculated. Student’s t test was used for independent samples when comparing a normally distributed quantitative variable between two groups. The normal distribution for Student’s t test was verified by the method of analysis of equality of variances (Folded F). Depending on the results of this analysis, the pooled method (variances with *p* ≥ 0.05) or the Satterthwaite method (variances with *p* < 0.05) were used. The chi-square test was used for categorical variables. The logistic regression model was used for unadjusted and adjusted odds ratio. The stepwise logistic regression model was used for adjusted analysis. The dependent variable was determined as the use or not of the drugs studied, and the independent variables selected in the model were those with *p* < 0.1 at bivariate analysis. The independent variables included in the model were marital status, education, previous traffic accidents, diabetes, high blood pressure, smoking, alcohol consumption, physical activity, hours of sleep, age, time in the profession, hours of driving, Epworth sleepiness questionnaire with values categorized as ≤10 and >10 points, blood glucose (categorized as ≤100 and >100 mg/dL), triglyceride levels (categorized as ≤150 and >150 mg/dL), total cholesterol (categorized as ≤200 and >200 mg/dL), BMI (categorized as <25 and ≥25 Kg/m^2^) and age. The significance level adopted for the statistical tests was 5% (*p* < 0.05). Statistical analyses were performed using the program SAS, University Edition^®^ (SAS Institute Inc., Cary, NC, USA).

## 3. Results

The demographic, occupational, clinical, and laboratory data and the use of illicit drugs of 2071 truck drivers who traveled on highways in the State of São Paulo from 2010 to 2016 are shown in Table 1. 

The mean age was 42.3 ± 11.1 years. They were mostly non-single (68.8%), with a predominant level of education up to 8 years of study (49.9%), corresponding to elementary school. 

The truck drivers have an average professional experience of 15.8 ± 11.3 years; 66.1% were hired by companies; 18.9% had at least one traffic accident; and 13.2% carried dangerous cargo. 

The total of 388 drivers who reported the use of illicit drugs at least once in their lives were mostly single and younger, with a higher level of education, less experience in the driving profession, a lower percentage of body fat, a lower prevalence of hypertension and greater use of alcohol and tobacco. 

The most prevalent self-reported drugs were marijuana (42%), amphetamine (39%) and cocaine (17%). In 52 (32%) drivers who declared themselves marijuana users, 43 (26%) also declared concomitant use of cocaine, and 9 (6%) used crack in addition to marijuana and cocaine. Among the 72 drivers who declared themselves current users (18.7%), the most prevalent drugs were marijuana (5.95%), amphetamine (4.7%) and cocaine (4.5%), which also appeared as combinations: cocaine and marijuana (2.2%), amphetamine and cocaine (0.4%), amphetamine and marijuana (0.3%) and amphetamine, cocaine and marijuana (0.2%).

**Figure 1:**
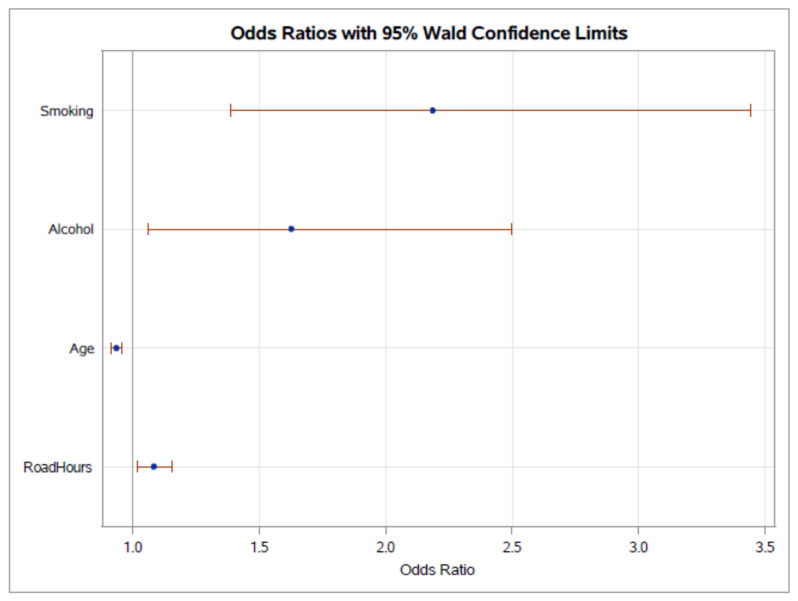
Odds ratio chart for the independent variables of smoking, alcohol consumption, age and hours of driving per day in the logistic regression analysis for the dependent variable, illicit drugs.

The logistic regression of unadjusted and adjusted odds ratio estimates is shown in Table 2. For the multivariable logistic regression analysis using illicit drugs as a dependent variable, we identified age (OR = 0.93 (95% CI: 0.91–0.95; *p* < 0.001)), smoking (OR = 2.18 (95% CI: 1.39–3.44; *p* = 0.001)), alcohol consumption (OR = 1.626 (95% CI: 1.06–2.49; *p* = 0.026)) and daily driving time (OR = 1.08 (95% CI: 1.01–1.15; *p* = 0.012)) as independent variables for illicit drugs (Figure 1).

## 4. Discussion

This study showed that the consumption of illicit drugs was higher in young drivers, smokers, and those who frequently consumed alcohol and who had a higher average number of daily driving hours. The prevalence of the use of illicit drugs and alcohol consumption found in the present study is within the average found in the literature for studies based on reports by truck drivers [8]. The percentages of consumption of amphetamine, marijuana and cocaine vary from 0.9% to 70%, 0.2 to 29.9% and 0.1% to 8.3%, respectively. This great variation between the study results is probably due to the methodological differences, sample variations and different geographic regions in which the research was carried out.

Amphetamine, known as “rebite” among Brazilian drivers, is used mainly as a stimulant to combat tiredness [9], as its main effects are increased alertness, decreased fatigue, elevated mood, euphoria and increased motor activity [21,22]. In Brazil, due to side effects, the potential to cause addiction and the tendency toward indiscriminate use, the sale, production and prescription of the substance has become prohibited in accordance with resolution RDC 52/2011 [23,24]. However, amid controversies among doctors and health agents, in 2017, a bill was approved for them to be produced, marketed and consumed under prescription again [25].

Leyton and colleagues point out that the legislation may have caused a change in the consumption patterns of amphetamine stimulants during the years in which it was in effect [26]. Between 2009 and 2016, the analysis of 4110 biological samples from drivers also showed an increase, although not statistically significant, in cocaine consumption. Amphetamine use changed over the years, with high consumption in 2010 (8.9%) but a decline in 2011 and 2012 (1.7% to 2.0%), followed by a stable period from 2013 to 2015 (ranging from 2.9% to 4.1%). In this research, it was found that such drugs were mainly used to combat fatigue in long-distance drivers, but the possibility of recreational use in drivers working shorter hours is not ruled out. During the period of this study (2010 to 2016), although the medication was prohibited, 33.33% of the drivers who declared consumption of other illicit drugs also reported the use of amphetamine as a stimulant. The amount is significantly higher than the 16.38% who reported having used amphetamine stimulants but did not report the use of other illicit drugs. Amphetamines exert peripheral sympathomimetic actions, producing elevated blood pressure, death of cardiac muscle cells, coronary insufficiency, increased heart rate and other cardiovascular changes resulting from increased concentration of norepinephrine in synapses [26].

Cocaine acts on the nervous system by inhibiting the reuptake of norepinephrine, dopamine and serotonin through interaction with its transporters, resulting in prolongation and overactivity of the sympathetic nervous system. Another possible effect is the blocking of sodium/potassium channels, causing cardiovascular changes, which may present increased and prolonged risks when there is an association between cocaine and alcohol [17]. In a review including a range of studies on the effects of the drug, it was found that its frequent use may be strongly associated with an increased risk of acute cardiovascular complications, such as hypertension, coronary aneurysms, arrhythmias and myocardial infarction [15,16,17,18,19,20,21,22,23,24,25,26,27].

Cocaine and its metabolites also affect behavior regarding food intake and suppress appetite, which can lead to the disruption of metabolic and neuroendocrine regulation, resulting in an increased risk of developing body weight problems, diabetes and metabolic syndrome [16]. Studies with humans have also shown anorexigenic effects of cocaine, and low caloric intake, together with abnormal metabolic and gastrointestinal functions, can lead to malnutrition among drug users [16].

In this study, body fat and hypertension were significantly related to the reported consumption of illicit drugs, and the lower percentage of fat for users of illicit drugs is consistent with what was found in the literature, since amphetamine and cocaine have proven effects on metabolic functions and are anorectic agents [16,23,27]. Although the prevalence of hypertension found among those who declared they had already used illicit drugs (9.8%) was lower than that found among those who had never used illicit drugs (15.2%), it should be considered that this factor may also be directly linked to a low percentage of body fat and the young average age.

According to the Brazilian cardiovascular prevention guidelines (BCPG), SAH is one of the most important risk factors for the development of coronary artery disease, heart failure, cerebral vascular disease, chronic kidney disease and atrial fibrillation [20]. The BGPC also show that 22 studies found a prevalence of SAH in the adult population of between 22.3% and 43.9% (average of 32.5%), being higher than the 50% prevalence identified in individuals between 60 and 69 years old and 75% in those over 70 years old; that is, there is a lower occurrence in younger individuals, and there is a visible progressive increase according to age.

Although there is evidence of an association between marijuana use and acute cardiovascular problems, it was not possible to assess the long-term chronic effects of using this drug [18]. The first study to link ischemic stroke in humans with marijuana use was done in the United States, and even after adjusting for other substances of abuse and eliminating confounding variables, it was found that the association persisted, showing that it is independent of other factors [16].

There is evidence that marijuana users who are also smokers are more likely to develop ischemic heart disease, a fact that was also reproduced by Singh and colleagues in a study review [28]. In view of the increase in drug use and the alarming number of new cardiovascular events related to it in recent decades, it is important to emphasize that there is a need to increase scientific work on this relation that is underestimated and underreported [28].

The variable “illicit drugs”, which had a positive association with the independent variables “age”, “smoking”, “alcohol consumption” and “marital status”, provided us with a profile of consumption that showed that younger drivers, with an average age younger than 40 years; singles; and individuals who smoke and consume alcohol are those most likely to use psychoactive substances. In addition, among drivers who reported having consumed substances of this type, the majority, when compared to non-users, had less time in the profession, drove for more hours during the day and traveled longer distances.

Girotto et al. reported similar results in truck drivers from the port of Paranagua, concluding that those most susceptible to the use of illicit psychoactive substances were those who were younger, who had irregular working hours, who were single and who normally traveled long distances [14]. Additionally, Bombana et al. found that drivers who travel in the main highways in the state of São Paulo and consume illicit drugs are younger, have less experience in the profession, and have a habit of driving for longer distances [13].

In our study, alcohol consumption and smoking were more prevalent among drivers who reported drug use at some point in life. This is in line with what was previously found in the literature [9]. In a study carried out in Japan, alcohol use appears to be associated with a younger average age, smoking, high blood pressure and a lower average BMI [29].

### Strengths and Weaknesses of the Study

This observational cross-sectional study individually estimated the effect of illicit drugs with respect to health and occupational risk factors. Our study used a large and population representative data of truck drivers that allowed a detailed analysis of the association between illicit drugs and demographic and cardiovascular risk factors. Nevertheless, due to its cross-sectional nature, the ability to elucidate causal relationships between the use of illicit drugs and health risk factors is limited.

The scarcity of studies targeting health-related risk factors of truck drivers is a limiting factor for queries and comparisons to be made with other percentages and prevalence values found prior to this research. There is a great need to develop new research in this sector, mainly in Brazil, where the population of truck drivers is large.

Another limitation is the dependence on the reliability of the interviewees’ report, who may omit information, especially with regard to drug use. On the other hand, when compared to studies based on toxicological tests, which may underestimate the use of illicit drugs because they only indicate the use of drugs in a short period prior to the examination, studies based on reports may be more comprehensive.

## 5. Conclusions

The use of illicit drugs was higher in young and single drivers and was associated with a higher prevalence of smoking, alcohol consumption and more daily driving hours, which are risk factors for NCDs, CVD and traffic accidents. Public policies to reduce drug use by these professionals can have an impact on reducing traffic accidents, as well as chronic degenerative diseases.

## Figures and Tables

**Table 1:** Sociodemographic data, occupational data, and clinical and laboratory data collected from 2071 truck drivers who traveled through the state of São Paulo between 2010 and 2016, classified according to the self-report of consumption of illicit drugs.

Illicit Drugs				
Variable	Total (*n* = 2071)	Used at Least once (*n* = 388)	Never Used (*n* = 1683)	|*p*|
Age (years)	42.27 ± 11.07	37.25 ± 9.45	43.43 ± 11.09	<0.0001
Marital status (%)				
Single	645 (31.2)	168 (43.3)	477 (28.4)	<0.0001
Not single	1423 (68.8)	220 (56.7)	1203 (71.6)	
Education level (%)				
Up to 8 years	1032 (49.9)	152 (39.2)	880 (52.35)	<0.0001
From 9 to 11 years	976 (47.2)	220 (56.7)	756 (45)	
Over 11 years	61 (2.9)	16 (4.1)	45 (2.65	
Type of contract (%)				
Self-employed	688 (33.3)	128 (33.1)	560 (33.35)	0.817
Hired	1366 (66.1)	258 (66.7)	1108 (66)	
Other	12 (0.6)	1 (0.2)	11 (0.65)	
Traffic accidents (%)	391 (18.9)	90 (23.2)	301 (17.9)	0.016
Dangerous cargo (%)	272 (13.2)	55 (14.2)	217 (12.9)	0.499
Sleeping hours (hours)	7.57 ± 1.54	7.4 ± 1.68	7.61 ± 1.51	0.027
Profession duration (years)	15.85 ± 11.3	12.36 ± 9.18	16.65 ± 11.59	<0.0001
Driving hours per day (hours)	9.07 ± 3.19	9.79 ± 3.33	8.91 ± 3.13	<0.0001
Distance travelled (km)	633.96 ± 850.03	798.19 ± 841.26	596.1 ± 847.79	<0.0001
Smoking (%)	474 (22.9)	129 (33.3)	345 (20.5)	<0.0001
Alcohol consumption (%)	969 (47.1)	207 (53.8)	762 (45.6)	0.003
Hypertension (%)	294 (14.2)	38 (9.8)	256 (15.2)	0.005
Diabetes (%)	131 (6.3)	17 (4.4)	114 (6.8)	0.080
Body mass index (kg/m^2^)	28.32 ± 5.01	28.28 ± 4.88	28.46 ± 5.52	0.310
Waist circumference (cm)	99.09 ±13.14	99.11 ±12.88	99.01 ± 14.24	0.898
Neck circumference (cm)	40.89 ± 4.23	40.84 ± 4.10	41.11 ± 4.75	0.291
Body fat (%)	24.32 ± 7.87	23.51 ± 8.17	24.5 ± 7.79	0.356
Glucose (mg/dL)	100.27 ± 42.96	97.67 ± 44.03	100.87 ± 42.70	0.199
Triglycerides (mg/dL)	199.74 ± 111.10	185.81 ± 102.60	202.53 ± 112.57	0.096
Cholesterol (mg/dL)	193.5 ± 33.35	190.02 ± 32.68	194.17 ± 33.46	0.172

**Table 2:** Logistic regression analysis of unadjusted and adjusted odds ratio and 95% Wald confidence limits of the data collected from 2071 truck drivers, classified according to the self-report of consumption of illicit drugs.

Variables	Odds Ratio Estimates					
	Unadjusted			Adjusted		
	Point Estimate	(95%) Wald Confidence Interval	|*p*|	Point Estimate	(95%) Wald Confidence Interval	|*p*|
Age	0.93	0.91–0.95	<0.001	0.94	0.93–0.95	*p* < 0.001
Marital status	0.71	0.59–0.85	0.001	0.76	0.48–1.22	0.257
Education level	1.59	1.31–1.94	<0.001	1.12	0.75–1.67	0.588
Traffic accidents	1.39	1.06–1.81	0.016	1.54	0.88–2.74	0.140
Sleeping hours	0.92	0.85–0.98	0.017	1.00	0.87–1.14	0.961
Profession duration	0.96	0.95–0.97	<0.001	1.00	0.96–1.04	0.987
Driving hours/day	1.09	1.05–1.13	<0.001	1.08	1.01–1.15	0.012
Smoking	1.94	1.52–2.47	<0.001	2.18	1.39–3.44	0.001
Alcohol consumption	1.39	1.11–1.73	0.004	1.63	1.06–2.49	0.026
Hypertension	0.61	0.42–0.87	0.006	0.94	0.44–1.99	0.864
Diabetes	0.63	0.37–1.06	0.083	1.17	0.33–4.15	0.802
Body mass index	0.92	0.72–1.18	0.520	1.02	0.97–1.06	0.467
Glucose	0.72	0.49–1.06	0.096	1.00	0.99–1.01	0.860
Triglycerides	0.67	0.47–0.96	0.030	0.66	0.41–1.05	0.467
Cholesterol	0.59	0.39–0.87	0.008	0.70	0.43–1.15	0.206

## Data Availability

The datasets used and/or analyzed during the current study are available from the corresponding author on reasonable request.
